# Effect of different composite core materials on fracture resistance of endodontically treated teeth restored with FRC posts

**DOI:** 10.1590/1678-77572016-0306

**Published:** 2017

**Authors:** Prapaporn PANITIWAT, Prarom SALIMEE

**Affiliations:** 1 Department of Prosthodontics, Faculty of Dentistry, Chulalongkorn University, Bangkok, Thailand

**Keywords:** Composite core material, Endodontically treated teeth, FRC post, Flexural modulus, Fracture resistance

## Abstract

**Objective:**

This study evaluated the fracture resistance of endodontically treated teeth restored with fiber reinforced composite posts, using three resin composite core build-up materials, (Clearfil Photo Core (CPC), MultiCore Flow (MCF), and LuxaCore Z-Dual (LCZ)), and a nanohybrid composite, (Tetric N-Ceram (TNC)).

**Material and Methods:**

Forty endodontically treated lower first premolars were restored with quartz fiber posts (D.T. Light-Post) cemented with resin cement (Panavia F2.0). Samples were randomly divided into four groups (n=10). Each group was built-up with one of the four core materials following its manufacturers’ instructions. The teeth were embedded in acrylic resin blocks. Nickel-Chromium crowns were fixed on the specimens with resin cement. The fracture resistance was determined using a universal testing machine with a crosshead speed of 1 mm/min at 135^0^ to the tooth axis until failure occurred. All core materials used in the study were subjected to test for the flexural modulus according to ISO 4049:2009.

**Results:**

One-way ANOVA and Bonferroni multiple comparisons test indicated that the fracture resistance was higher in the groups with CPC and MCF, which presented no statistically significant difference (p>0.05), but was significantly higher than in those with LCZ and TNC (p<0.05). In terms of the flexural modulus, the ranking from the highest values of the materials was aligned with the same tendency of fracture loads.

**Conclusion:**

Among the cores used in this study, the composite core with high filler content tended to enhance fracture thresholds of teeth restored with fiber posts more than others.

## Introduction

The use of fiber reinforced composite (FRC) posts has tremendously increased the restoration of endodontically treated teeth due to their favorable physical properties, such as high tensile strength and good fatigue resistance. Also, their modulus of elasticity is similar to that of dentin. In combination with FRC post, the composite core build-up material is often used to restore the coronal portion of the teeth and to achieve retention and resistance form for the crown^4,7^. The clinical evaluation of the FRC post and core restoration has reported high success rate with reduction in failure of root fracture. The common failures of these restorations related to materials are post debonding, crown debonding, or post fracture, which are usually combined with core failure, especially in teeth with few coronal walls^21,27^. Since the FRC post is flexible, the core build-up material must be strong enough and resist multidirectional masticatory force. Furthermore, it has to withstand a process of crown preparation by rotary cutting instrument. Therefore, the core material is also a critical component for the overall success in the restoration of endodontically treated teeth, especially when using it with FRC post.

Resin composite is a popular core build-up material to be used with FRC post due to similarity to tooth structure in hardness and fracture toughness, giving the ability to perform the preparation after curing. Restorative composites can be regularly employed for core build-up material^4,7,21,27^. Nowadays, there are many resin composites that are specifically designed for core build-up with increase in fillers for higher strength and enhance for easy manipulation. These materials are different in amount and types of filler, viscosity, curing mode, build-up technique, among others, while their physical properties have been investigated in many aspects^11,20,22,28^. Regarding viscosity, high viscosity composite core materials are handled by using incremental technique to ensure complete polymerization and optimal strength. Low viscosity core build-up composites are generally prepared in an automix syringe that can avoid air contamination. These materials can also be used for cementing the FRC post and core material at the same time. In order to allow polymerization in the root canal, they are dual-curing composites which are preferable to use with fiber posts, where light curing might not be perfectly completed. It can be noticed by clinicians that low viscosity core materials are more easily prepared by diamond cutting instrument than high viscosity materials. Thus, the low viscosity composite core might be easy to handle, but the strength might be reduced. Rüttermann, et al.^22^ (2011) investigated the physical properties of direct core materials and found that the flexural strength of high viscosity composites (Clearfil and MultiCore HB) is higher than that of flowable composites (Rabilda SC). However, some studies have shown that low viscosity composite core materials had higher bond strengths to FRC post than hybrid composites^23,25^. The study of Naumann, et al.^17^(2010) showed no significantly higher risk of failure between high viscosity composite (Clearfil Core), low viscosity composite (LuxaCore Dual), and self-adhesive cement (RelyX Unicem) for core build-up after long term storage, thermocycled and mechanically loaded. This may indicate that the strength of core material alone might not influent the strength of endodontically treated teeth restored with FRC post. The study of Kim and Lee^12^ (2012) investigated the use of various posts and cementation procedures and showed that the change of post or core did not influence the fracture strength and failure patterns. However, various factors took part in the results of the study, since some core materials were used both as cement and core material.

This study aimed to evaluate the fracture resistance of endodontically treated teeth restored with FRC post, using four different core build-up composite materials: one restorative nanohybrid composite (Tetric N-Ceram (TNC)) and three specified composites for core build-up materials (Clearfil Photo Core (CPC), MultiCore Flow (MCF), and LuxaCore Z-Dual Automix (LCZ). The null hypothesis was that there would be no difference in the fracture load of the restorations among these composites.

## Material and methods

### Fracture resistance test

#### Specimen pre-treatment

Forty extracted human lower first premolars, due to orthodontics treatment protocol, with similar form and size of roots, were selected. Each tooth was visually inspected to be free of cracks, dental caries, restorations, or other defects. The dimensions of the teeth were measured mesiodistally, labiolingually, and in root length, using a digital vernier caliper (Mitutoyo, Kawasaki, Japan). Teeth size with 5.5+0.5 mm mesiodistally, 8.0+0.5 mm labiolingually, and 14.5+0.5 mm in root length were chosen. All teeth were cleaned and debrided of soft tissues and stored in 0.9% normal saline until used.

#### Root canal preparation

The clinical crowns were decoronated perpendicularly to the root axis 1 mm above the cementoenamel junction (CEJ) by a low speed cutting machine (ISOMET 1000; Buehler Ltd, IL, USA). The pulpal tissue was removed with a barbed broach and a stainless steel K-file size 15 (Dentsply Maillefer, Ballaigues, Switzerland) was inserted into the canal through the apex to establish the working length by subtracting 1 mm from this measurement. All teeth were endodontically treated using a step-back technique with master apical file size 40 and coronal flaring size 70. During instrumentations, the root canals were irrigated with 2.5% NaOCl, alternating irrigation with 2.5% NaOCl and 17% ethylenediaminetetraacetic acid solution, and final irrigation with normal saline. The canals were dried and then obturated using a lateral condensation technique with gutta-percha cones and eugenol-contained root canal cement (CU dental Product, Bangkok, Thailand)^5^. The excess of gutta-percha was removed with a hot instrument and sealed with provisional filling material (Cavit; 3M ESPE, Seefeld, Germany) to a depth of 3 mm. All specimens were stored at 37^0^C during 24 h for complete setting of cement^14^.

#### Specimen’s block preparation

After endodontic treatment, a D.T. universal drill was used to prepare a post space to a depth of 10 mm, leaving 4 mm of intact gutta-percha as the apical seal. The canals were then shaped with D.T. finishing drill corresponding to the post (D.T. Light-Post Illusion size 1; RTD, Saint-Egreve, France) with a coronal diameter of 1.5 mm and 0.9 mm at its apical tip. A vinyl polysiloxane (Reprosil light body consistency; Dentsply Caulk, USA) was used to simulate the periodontal ligament. Each root was dipped into melted modeling wax at 2 mm below the CEJ, which allowed the thickness of approximately 0.2 mm. The tooth was then attached to a surveyor (Dentalfarm, Torino, Italy) with the D.T. Light drill in the canal and placed in a plastic mold (22 mm in diameter and 20 mm in height) 2 mm coronally above the upper edge. The autopolymerizing acrylic resin (Formatray; Kerr Corporation, Orange, CA, USA) was poured in the mold, resulting in root embedded in acrylic resin base. A silicone mold index was made as an aid for the specimen repositioning when replacing the wax with vinyl polysiloxane.

#### Post and core build-up procedure

**Figure 1:**
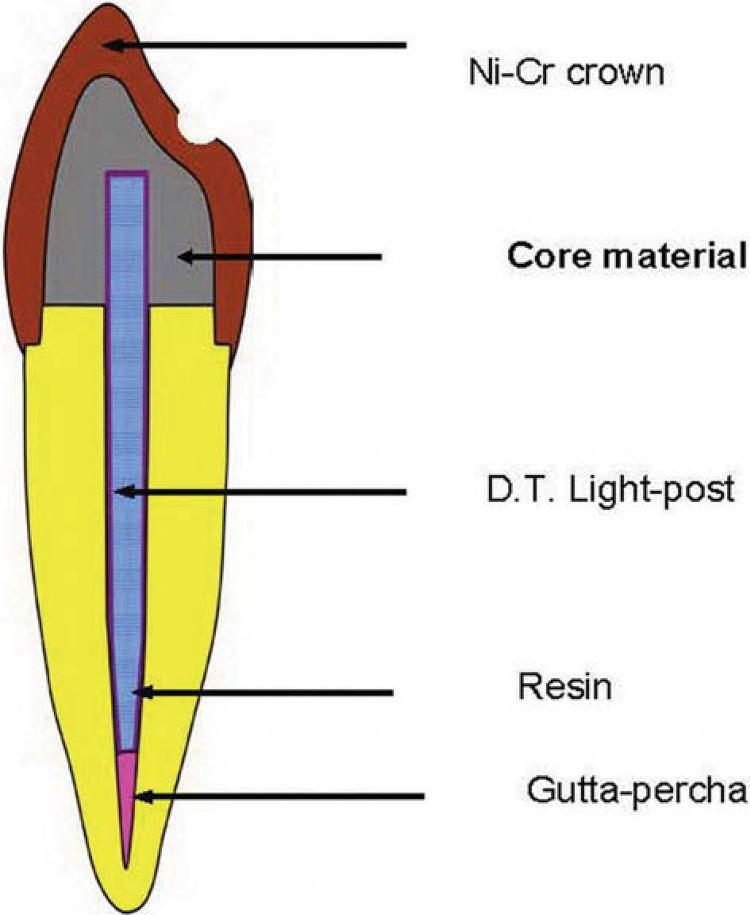
Schematic illustration of tooth specimen with restoration

**Figure 2:**
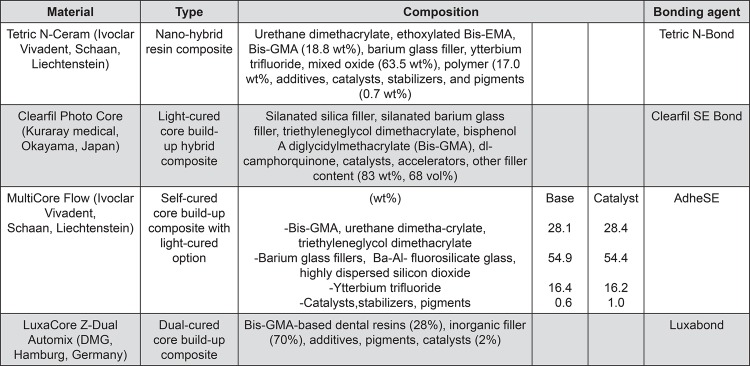
Core build-up materials used in this study, their compositions and recommended bonding agent

All specimens were then randomly divided into four groups of ten samples according to different composite core materials to build-up (Figure 1). The core materials used and their compositions are shown in Figure 2. Each fiber post was cut at 14 mm length with a high-speed diamond rotary cutting instrument (ISO 314197; Intensiv SA, Grancia, Switzerland). At this length, 10 mm of the post would be in the root, leaving 4 mm projecting above the prepared tooth. Surface treatment of post was performed with silane coupling agent (mixture of Clearfil SE bond primer and porcelain bond activator; Kuraray Medical). The root canal was irrigated with normal saline, dried with paper points, and conditioned with self-etching primer (ED primer II A&B; Kuraray Medical) for 30 seconds, followed by gently air dried. The post was cemented with dual-polymerizing resin cement (Panavia F2.0; Kuraray Medical) according to the manufacturer’s instruction. The excess cement was removed and the post was cured with a light-polymerized unit (Elipar Trilight; 3M ESPE, St. Paul, MN, USA) for 20 seconds *per* surface. Before core build-up procedure, a dentin bonding agent was applied to dentin according to the manufacturers’ recommendations (Table 1). The core build-up was then continued using a transparent matrix band. For TCN and CPC, the incremental technique of 2-mm layer was performed per 40 seconds of light curing. For MCF and LCZ, the material was injected around the post and light cured for 40 seconds. After finished, the core was prepared for full metal crown with a circumferential 0.5 mm chamfer finishing line at CEJ level. The height of the core was 6 mm facially and 3 mm lingually above the CEJ. This resulted in the abutment height at 5 mm of core material and 1 mm ferrule. The coronal end of the post was completely covered with the resin core 1 mm^6^. All measurements were done by digital caliper.

**Table 1:** Mean and standard deviations of the flexural modulus of the four groups

Group (n=5)	Mean ± SD (GPa)
Clearfil Photo Core (CPC)	17.00 ± 2.33
MultiCore Flow(MCF)	7.20 ± 0.36
LuxaCore Z-Dual Automix(LCZ)	6.67 ± 0. 54
Tetric N-Ceram(TNC)	5.90 ± 1.04

#### Crown restoration

An impression of the restoration was made with vinyl polysiloxane impression material and poured with type IV dental stone (Vel-Mix; Kerr Corporation, Orange, CA, USA). The full metal crown of each specimen was made by creating the wax pattern with blue inlay casting wax (Kerr Corporation) on the dies with the same outer contour. A notch was prepared for testing on the center of the occlusal surface. Each pattern was invested and casted using Nickel-Chromium alloy (4all Williams #0123; Ivoclar Vivadent). The crown was finished, polished and the fit to the specimen was evaluated by visual inspection and checked with vinyl polyether silicone (Fit checker; GC Corporation, Tokyo, Japan). The crown was then cemented with Panavia F 2.0. The procedure was performed by conditioning dentin with ED primer for 30 seconds and cementing the crown to the core. Each surface was light polymerized for 20 seconds. An oxygen barrier (Oxyguard II gel; Kuraray Medical) was applied to the margin of the crown for 3 minutes, and then the excess cement was removed. The specimens were stored at 37^0^C 24 h prior to testing.

The fracture resistance test was performed by a universal testing machine (model 8872; Instron Ltd, High Wycombe, UK) at 135^0^ to the long axis of the tooth. The load tip was placed on the prepared occlusal notch. A continuous compressive force was applied at a crosshead speed of 1 mm/min until failure. The highest fracture load of each specimen was measured by a sudden drop in load magnitude, recorded in Newton. All specimens were visually examined under a stereomicroscope (ML9300; Meiji, Tokyo, Japan) for the mode of failure.

## Flexural modulus test

All resin composite core build-up used in this study was tested for flexural strength, according to ISO 4049:2009 (E)^10^. Preparations of a beam-shaped specimens (n=5) were performed according to the manufacturer’s recommendation, using a stainless steel mold (25×2×2 mm^3^). Each specimen was light cured for 60 seconds *per* side. All specimens were stored at 37±1°C for 24 hours in distilled water prior to testing. The flexural strength was determined by using a universal testing machine at a crosshead speed 0.75±0.25 mm/min until the fracture occurred. The highest load of each specimen was recorded. The flexural strength and flexural modulus were calculated from the following equations:


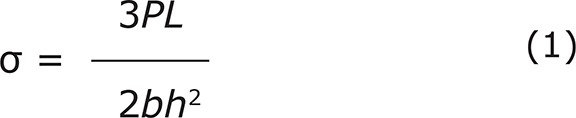



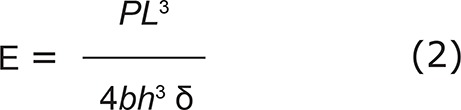


Where σ was the flexural strength (MPa), E was the flexural modulus (MPa), P was the maximum load (N), L was the length of span between the supports (mm), b was the width of the specimens (mm), h was the height of the specimens (mm), and δ was the deflection (mm).

## Data collection and analysis

The data were analyzed using statistical software (SPSS Statistics 17.0; SPSS Inc, IL, USA). The normal distribution was tested with Kolmogorov-Smirnov and Shapiro-Wilk tests. One-way analysis of variance (ANOVA) and Bonferroni multiple comparisons test were used to analyze the differences between groups (α=0.05), respectively.

## Results

**Figure 3:**
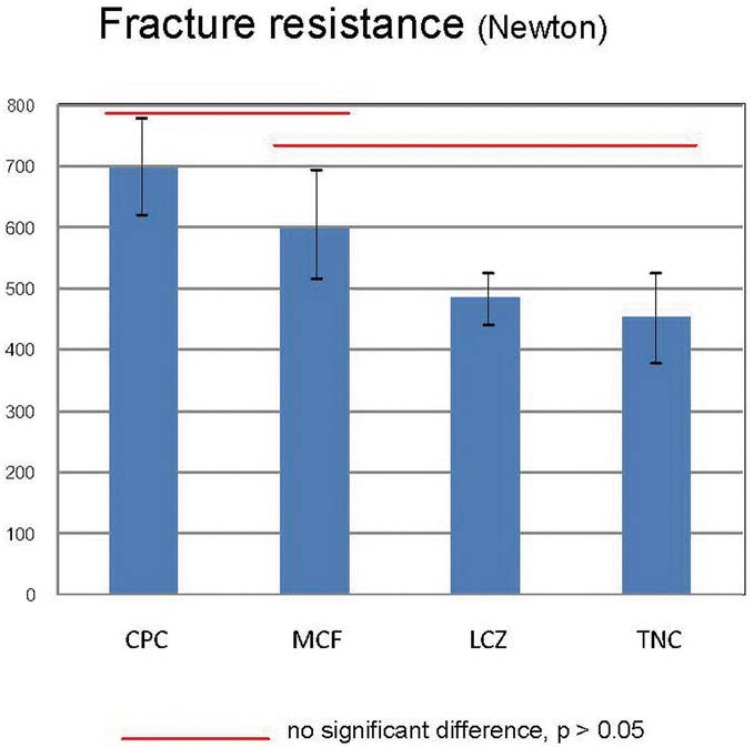
Results of fracture resistance load in the four study groups

**Figure 4:**
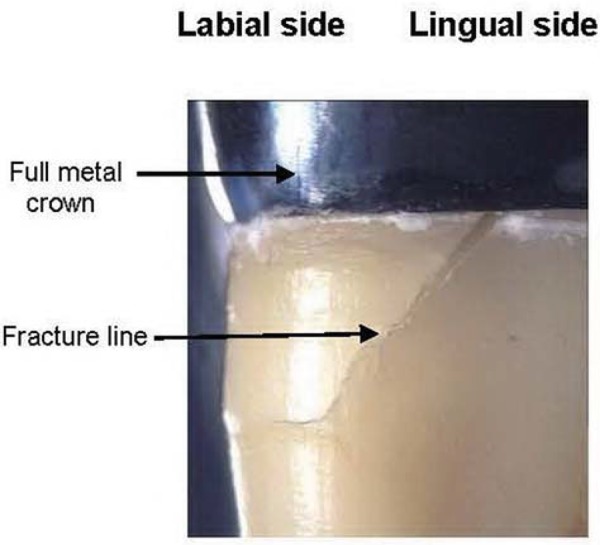
Fracture pattern observed under stereomicroscope (x10)

The fracture load was higher in the group with CPC (699.1±173.4 N), followed by the groups with MCF (587.1±182.3 N), LCZ (483.1±80.5 N), and TNC (451.4±154.5 N), respectively. ANOVA and Bonferroni multiple comparisons test showed that the fracture resistance for CPC was not significantly different from MCF (p>0.05), but significantly higher than that of LCZ and TNC (p<0.05) (Figure 3). When the specimens were examined under the stereomicroscope, the pattern of failure for all groups originated at the lingual margin of the crown and continued obliquely in an apical-facial direction, as shown in Figure 4.

The results of flexural modulus of the core materials are shown in Table 1. The ranking of these values align with the same trend of the fracture resistance, in which the highest modulus experienced the highest value of fracture resistance.

## Discussion

In material aspect, the failure of restoration of endodontically treated teeth using FRC post and core concerns many factors, such as types of FRC post, shape of post, surface treatments, cementation, and core material used. In this study, we aimed to investigate the effect of the core material used by using fracture resistance test. Therefore, the other factors were controlled for the same condition, except the types of core material.

Due to the statistical analysis, the null hypothesis was rejected, since there were significant differences among fracture resistances in the group using different composite core build-up materials. The fracture resistance load of CPC and MCF groups was significantly higher than that of LCZ and TNC groups. The results of this study confirmed the previous studies of Bitter, et al.^3^(2016), Naumann, et al.^18^(2011), and Rüttermann, et al.^22^(2011), in which Clearfil Core showed a mechanical load resistance higher than other materials. Although core material was the only variable factor, the differences in fracture loads could be the properties of core material concerning strength, rigidity, bonding ability to post and dentin, polymerization modes, etc. The strength of core materials is one of the important properties in obtaining a long term success of restoration, especially when the remaining tooth structure is limited^1^. In this situation, stress was placed on the core material that demands a higher strength material to resist a fracture load. Composite materials are generally composed of organic polymer matrix, a compound of Bis-GMA and filler particles. TNC is a conventional nanohybrid composite, and the nano-sized filler improves its good physical properties and esthetics^9^. The other materials are resin composites specifically used for core build-up. CPC is a light-curing hybrid composite with high translucency that show high depth of cure to 7 mm. MCF and LCZ are dual-curing composites (with fluoride filler and zirconium dioxide filler, respectively) that have low consistency, which allows the mixing and application in root canal. According to the manufacturers’ information, CPC has the highest filler content (83 wt%), followed by MCF (base 71.3 wt%, catalyst 70.6 wt%), LCZ (70 wt%), and TNC (63.5 wt%), respectively. It can be noticed that the results of fracture resistance load had the same trend with the filler content in core materials.

In this study, we also investigated the flexural modulus of the core material used. The results showed the same trend to the fracture resistance. This may confirm that increased filler content results in a higher flexural modulus, and the higher modulus the composite core material had, the more fracture resistance increased. These results agree with the previous studies of Ahn and Sorensen^1^ (2003), which noted that CPC showed significantly higher value in flexural strength, shear bond strength, and fracture toughness compared to other core materials. The flexural modulus indicates the relative stiffness of the material within an elastic range that reflects the strength and longevity of the restoration. The desired modulus of core materials should be similar to that of dentin to uniformly distribute the masticatory forces to the post and root. Similar moduli minimize the interfacial stress between two different materials^29^.

The bonding ability of the core material is also the factors concerning the results. Two main bonding surfaces have cooperated: core/dentin interface at the cross section 1 mm above the CEJ of the tooth and core/post interface along the core height coronally. Although the area of core/dentin bonding surface was small, the position was near to the fulcrum, which was in a critically area. This study used dentin bonding agent recommended by each manufacturer before core build-up procedure to avoid incompatibility between materials. The study of O’Keefe and Powers^20^(2001) showed incompatibilities between a self-cured core material and a dual-cured adhesive. Their study also showed that Clearfil SE Bond had higher bond strengths than Prime & Bond NT when bonded to the same core material. This might help increasing the high fracture resistance in CPC groups apart from the effect of fillers content. CPC, which is a light-curing composite core material, also showed higher bond strength to dentin and higher flexural strength than chemical and dual-curing composites^1^.

At the post/core material interface, the surface treatment of the posts was performed with silane coupling agent from the same manufacturer of CPC. A previous study showed that low viscosity core materials have higher microtensile bond strength to FRC post than conventional composites using incremental technique^25^. Flowable composites achieved better integration with fiber post since bubbles and voids within the core or the core/post interface are minimized^15^. The low consistency of the core material such as MCF and LCZ might be easier to lute the surface of the post, with less air contamination, providing higher bond strength. For TNC and CPC, which use incremental technique, it can be noticed that CPC, which is less consistent, was more translucent and may have allowed less void, promoting more complete polymerization^13^. It seemed that the curing mode did not affect the results, since CPC showed the highest and TNC showed the lowest values of fracture resistance. The effective bond between the post and the core material should be further investigated to enhance higher strength in endodontically teeth with more coronal structure loss.

Surface treatment of the FRC post in this study was also performed by using mixture of Clearfil SE bond primer and porcelain bond activator, which come from the same manufacturer of CPC. This was to improve the adhesion of the post and cement interface chemically. The mechanical treatment of airborne-particle-abrasion can improve bond strengths, but its technical sensitivity leaded to a risk of modifying the shapes and fit of the post^24^. The compatibility of the materials used may have affected the results. In the CPC group, core material, bonding agent (Clearfil SE Bond), and resin cement (Panavia F2.0) are from the same manufacturer, which might be more compatible than the other groups.

Regarding mode of failures, the fracture pattern of all groups was similar. The direction of the force applied obliquely on the occlusal surface of the simulated crown may cause the post to flex labially^8^. This generates a compressive stress in the labial dentin while the lingual dentin is under tension. The fulcrum was located at the upper border of the acrylic block, simulating the labial alveolar bone crest. Tension forces might cause an adhesive failure of the post-cement-root dentin interface. Then, the post might be loose within the root canal and consequently act like a wedge. Loads exceeding the tensile strength of dentin leaded to cervical root fracture, oblique from the cervico-lingual to labio-apical direction^16^. This finding agrees with a three-dimensional finite element analysis, in which stress concentration in the post region was observed at the interface between lingual side of fiber post and resin core, and maximum stress in the remaining radicular dentin was on the inner side of the proximal wall at cervical level^19^. The fracture pattern observed indicated higher stress concentrations developed in the coronal third of the dentin than at the apex. Furthermore, high stress concentrations increased between a rigid and less rigid part in the cervical area of the metal crown margin and the brittle dentin. In contrast, the placement of all-ceramic or porcelain fused to metal crowns might have different results from the full metal crown used in this study, due to different mode of failure^26^.

Ferrule length and remaining coronal dentin play important roles in the success of endodontically treated teeth. The absence of coronal wall might increase the risk of restoration failure^30^. The study of Akkayan^2^ (2004) showed no significant difference between 1 and 1.5 mm ferrules length in specimens restored with quartz fibers post and resin composite core. Thus, a 1-mm ferrule height was prepared in this study to simulate the condition that can be retained with FRC post, while the effect of the core material used can be clearly investigated.

Various material properties concern the success of endodontically treated restorations. The previous study concluded that the performance of the core materials depended on their formulation, as well as on their proper curing process^22^. Since there are many restorative products in the market nowadays, clinicians should consider selecting a restorative material not only by the easiness of use, but also by its suitable properties to gain more successful restorations. Thus, it is important to select the suitable composite core matereial to use with the FRC post, especially when the tooth has moderate or severe coronal structure loss.

The static loading is a standard test in the material evaluation process and is commonly used to obtain information about the potential for clinical success. For imitating clinical situations, thermal cycling or fatigue testing may be further appropriated.

## Conclusions

Within the limitations of this study, it was shown that resin composite core build-up material with higher filler content tended to enhance more fracture resistance for endodontically treated teeth restored with FRC post.
